# Lysophosphatidic acid stimulates epithelial to mesenchymal transition marker Slug/Snail2 in ovarian cancer cells via Gαi2, Src, and HIF1α signaling nexus

**DOI:** 10.18632/oncotarget.9224

**Published:** 2016-05-07

**Authors:** Ji Hee Ha, Jeremy D. Ward, Rangasudhagar Radhakrishnan, Muralidharan Jayaraman, Yong Sang Song, Danny N. Dhanasekaran

**Affiliations:** ^1^ Stephenson Cancer Center, The University of Oklahoma Health Sciences Center, Oklahoma City, Oklahoma, USA; ^2^ Department of Cell Biology, The University of Oklahoma Health Sciences Center, Oklahoma City, Oklahoma, USA; ^3^ Cancer Research Institute, Seoul National University, Seoul, Korea

**Keywords:** ovarian cancer, EMT, LPA, HIF1α, metastasis

## Abstract

Recent studies have identified a critical role for lysophosphatidic acid (LPA) in the progression of ovarian cancer. Using a transcription factor activation reporter array, which analyzes 45 distinct transcription factors, it has been observed that LPA observed robustly activates the transcription factor hypoxia-induced factor-1α (HIF1α) in SKOV3.ip ovarian cancer cells. HIF1α showed 150-fold increase in its activation profile compared to the untreated control. Validation of the array analysis indicated that LPA stimulates a rapid increase in the levels of HIF1α in ovarian cancer cells, with an observed maximum level of HIF1α-induction by 4 hours. Our report demonstrates that LPA stimulates the increase in HIF1α levels via Gαi2. Consistent with the role of HIF1α in epithelial to mesenchymal transition (EMT) of cancer cells, LPA stimulates EMT and associated invasive cell migration along with an increase in the expression levels N-cadherin and Slug/Snail2. Using the expression of Slug/Snail2 as a marker for EMT, we demonstrate that the inhibition of Gαi2, HIF1α or Src attenuates this response. In line with the established role of EMT in promoting invasive cell migration, our data demonstrates that the inhibition of HIF1α with the clinically used HIF1α inhibitor, PX-478, drastically attenuates LPA-stimulates invasive migration of SKOV3.ip cells. Thus, our present study demonstrates that LPA utilizes a Gαi2-mediated signaling pathway via Src kinase to stimulate an increase in HIF1α levels and downstream EMT-specific factors such as Slug, leading to invasive migration of ovarian cancer cells.

## INTRODUCTION

Ovarian cancer remains as the most fatal gynecological cancers in the world with a five-year survival rate of only approximately 45% [1]. This is primarily due to our poor understanding of the disease in addition to the asymptomatic nature of this cancer in the early stages. LPA is known to elicit its diverse cellular responses by stimulating various members of the G protein families Gi, G12, and Gq [2–6]. Importantly, studies from several laboratories, including ours [7–9], have shown that LPA plays a crucial role in the progression of ovarian cancer [10–12]. Indeed, our lab and others have shown that LPA-mediated signaling stimulates proliferation, migration, and invasion of ovarian cancer cells [5, 7–9, 13]. Increased levels of LPA in the ascites of the ovarian cancer patients and a robust membrane-bound LPA-synthetic machinery quite adjacent to LPA-receptors in ovarian cancer cells raise the concentration of LPA in the tumor microenvironment to micromolar concentrations, which may not allow LPA-receptor antagonist to be used as an effective therapeutic agents. Therefore, defining a signaling node downstream of LPA-receptors has become critical to develop therapeutic strategy for inhibiting LPA-mediated oncogenic pathway(s). With this overarching goal, our laboratory as well as others have shown that the oncogenic activity stimulated by LPA involves the *gep* oncogenes Gα12 and Gα13 [14] as well as the putative *gip2* oncogene Gαi2 [8, 15]. However, the role of these oncogenic Gα-subunits in the activation of specific LPA-mediated oncogenic responses is far from clear. Therefore, we focused on defining the signaling nodes involved in LPA-mediated activation of a specific transcription factor, if any, which can be correlated with a critical oncogenic response.

HIF1α has been shown to play a critical role in ovarian cancer malignancy, especially ovarian cancer cells found in the hypoxic conditions of the peritoneal cavity [16–18]. While HIF1α is rapidly degraded in normoxia, it is rapidly stabilized by hypoxia, thereby promoting its transcriptional activity [19, 20]. In addition to hypoxia, several growth factors including LPA have been shown to induce the expression/stability of HIF1α [21–24]. However, the mechanisms by which LPA stimulates the increase in the levels of HIF1α and its activation are not fully understood.

The activation of HIF1α involves its dimerization with the constitutively expressed HIF1β [25]. This is followed by the translocation of HIF1α and HIF1β dimers to the nucleus and subsequent HIF1α mediated transcription of a multiple genes that can promote angiogenesis, glucose metabolism, cell survival, proliferation, and metastasis in cancer [26]. Importantly, one of the critical oncogenic responses orchestrated by HIF1α is epithelial-to-mesenchymal transition (EMT) process [27–29] in which the cancer cells switch expression of markers of epithelial cells, such as E-cadherin to mesenchymal markers such as N-cadherin, vimentin, and transcription factors Snail1, Slug (Snail2), ZEB1, ZEB2 and Twist thereby facilitating the invasive migration and metastasis of cancer cells [28, 29]. Cells suppress the expression of proteins such as E-cadherin that allow for cell-to-cell attachment and increase the expression of proteins such as N-cadherin and vimentin that promote cell-detachment and migration. Furthermore, expression of EMT-specific transcription factors has been shown to increase the expression of proteins that can degrade extracellular components, which allow the cancerous cells to invade neighboring tissues [30]. This change in cellular markers characterizes a specific shift in the phenotype of the cancerous cells from being stationary to markedly increased invasive phenotype [28, 29]. Accordingly, EMT has been well recognized as a critical mechanism underlying carcinogenesis, cancer progression, and metastasis. Therefore, identifying pathways that can inhibit EMT are of critical importance for cancer therapy.

In the present study, using a transcription array to identify transcription factors activated by LPA-mediated signaling, we demonstrate that LPA potently stimulates the activation of HIF1α via a pathway involving Gαi2 and Src. We further demonstrate that that the activation of LPA-Gαi2-Src-mediated signaling pathway induces EMT in ovarian cancer cells and subsequent invasive migration of ovarian cancer cells that can be inhibited by PX-478, a clinically tested inhibitor of HIF1α. Thus, our current study demonstrates that LPA stimulates a signaling nexus involving Gαi2, Src, and HIF1α to induce EMT and migration of ovarian cancer cells. Furthermore, we show that Gαi2 signaling is necessary and sufficient for hypoxia-mediated induction of HIF1α expression, which has not been shown, to our knowledge, by any previous studies to date.

## RESULTS

### LPA stimulates the activity and expression of HIF1α in ovarian cancer cells

**Figure 1:**
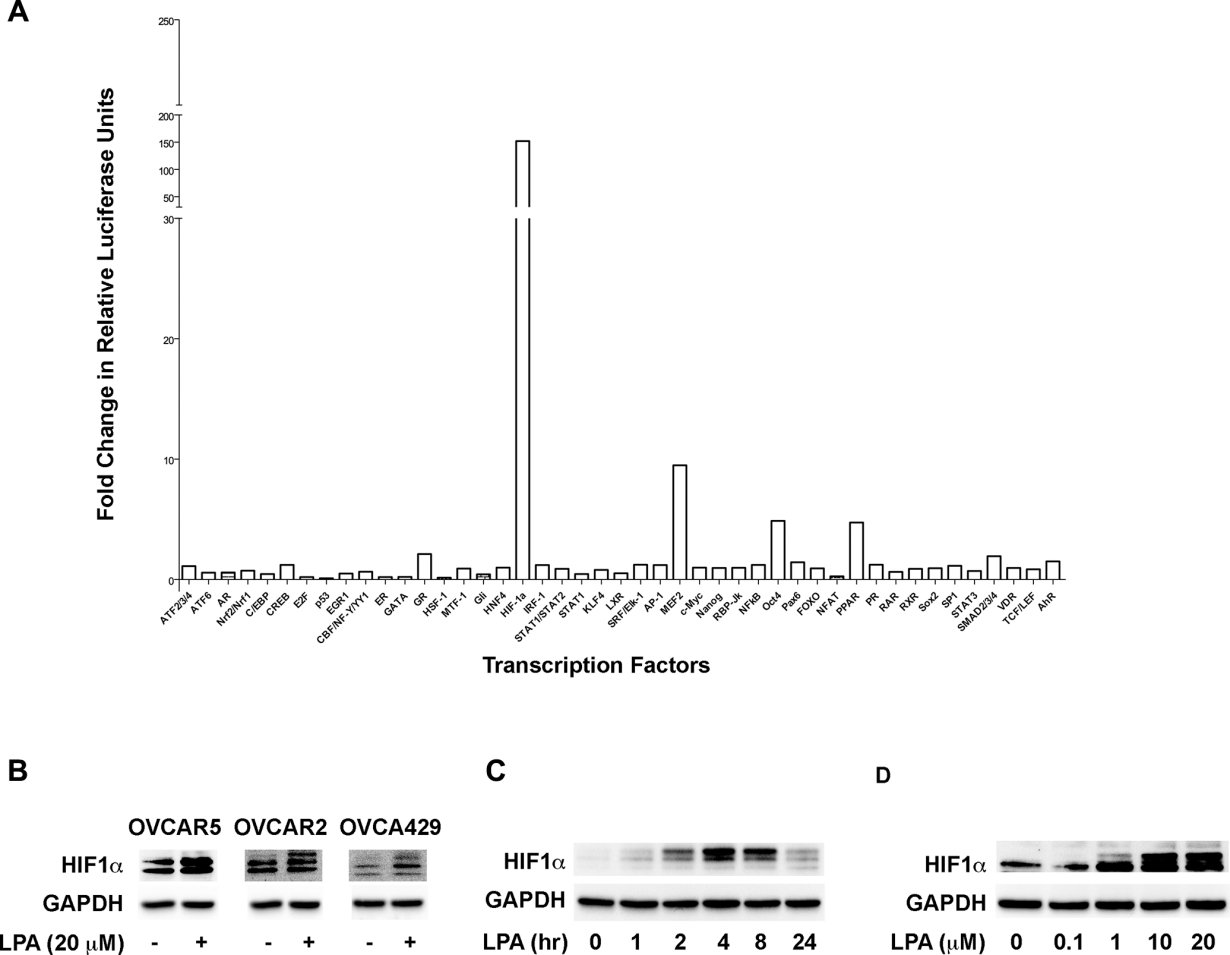
LPA stimulates the activity and the expression of HIF1α (**A**) LPA stimulates the activation of HIF1α. SKOV3.ip cells were stimulated with 20 μM of LPA for 20 minutes or left untreated in serum-free condition. Activation profiles of 45 different transcription factors were analyzed with a Cignal^™^ 45-Pathway Reporter Array per manufacturer's protocol. (**B**) LPA-stimulated increase in HIF1α is independent of cell types. OVCAR5, OVCAR2, and OVCAR49 cells were serum-starved overnight and then stimulated with 20 μM of LPA for 4 hours. The cells were lysed and the level of HIF1α was analyzed via Western blot. GAPDH was used as a loading control for each lane (*n* = 3). (**C**) Time-course Analysis of LPA stimulated increase in the levels of HIF1α. SKOV3.IP cells were serum-starved overnight for 16 hours following which they were stimulated with 20 μM of LPA for the indicated time points. Lysates were subjected to immunoblot analysis using antibodies to HIF1α. The blot was stripped and re-probed with antibodies to GAPDH to ensure equal loading of proteins in each lane. (**D**) LPA-stimulated increase in the levels of HIF1α is dose-dependent. SKOV3.IP cells were serum starved overnight and then stimulated with the different concentrations of LPA for 4 hours. The cells were lysed and subjected to immunoblot analysis using antibodies to HIF1α. The stripped blot was probed with GAPDH-antibodies to monitor equal loading of proteins.

In order to identify possible mechanism utilized by LPA to drive the progression of ovarian cancer we employed a transcription factor array that can analyze the activation profile of fortyfive different transcription factors. SKOV3.ip cells were stimulated with LPA for 20 minutes along with the appropriate vehicle control and the lysates were subjected to the transcription array analysis. Our results indicated that LPA stimulation activated several transcription factors that have previously been shown to be stimulated by LPA including STAT3 [7, 31, 32] and CREB [7, 33, 34], thus establishing the functional validity of our array analysis. In addition, we observed that LPA stimulated the activity of HIF1α by 150-fold compared to the untreated control cells and its activation far exceeded the activation of any other transcription factor on the array (Figure 1A). In light of the recent findings that HIF1α plays a critical role of in ovarian cancer progression and malignancy [16–18], we sough to investigate the mechanism by which LPA stimulates the activity of HIF1α. Since the expression levels of HIF1α correlate with its activation [25, 35, 36], we first determined the expression levels of HIF1α following LPA stimulation in a panel of ovarian cancer cells. Our results indicated that LPA stimulated an increase in HIF1α in three different ovarian cancer cell lines, namely OVCAR5, OVCAR2, and OVCA429 (Figure 1B). Thus, our results establish that the effect of LPA on HIF1α is cell-line independent. Next, we carried out a time-course analysis for the expression of HIF1α in SKOV3.ip cells in response to LPA. As shown in Figure 1C, LPA stimulated increase in the levels of HIF1α could be seen from 60 minutes onwards. Furthermore, it can be observed that the levels of HIF1α increases with time, reaching the maximum levels by 4 hours. Next, we carried out a dose-response curve with different concentrations of LPA. Our results indicated that LPA stimulated an increase in HIF1α levels in a dose-dependent manner (Figure 1C). Since the maximal increase of HIF1αcould be seen with 10 μM LPA by 4 hours, the remainder of the experiments in this study involved the use of 10 μM LPA.

### LPA signaling to HIF1α involves Gαi2

**Figure 2:**
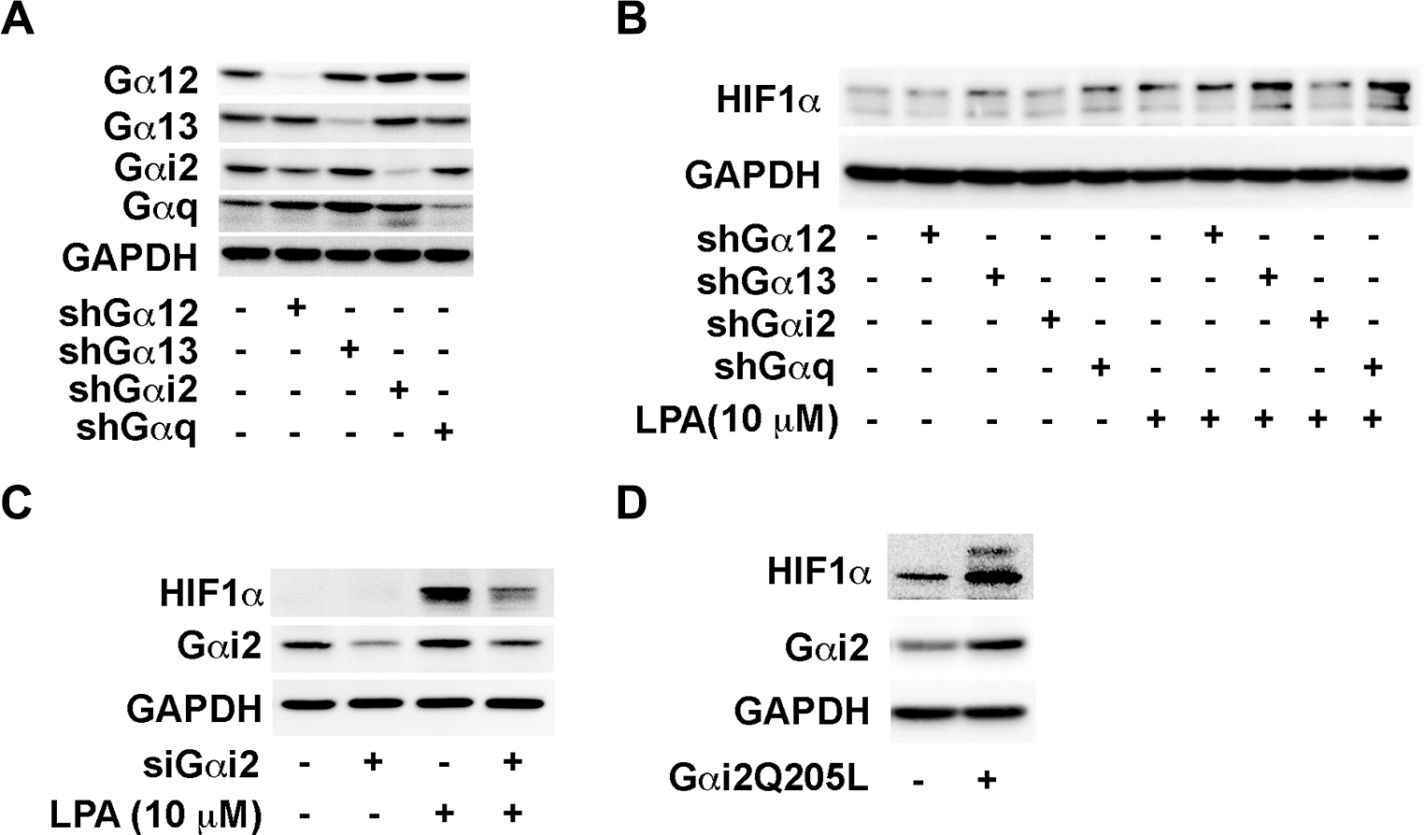
LPA stimulates an increase in the levels of HIF1α via Gαi2 (**A**) Confirmation of knockdown of G proteins in SKOV3.ip stable clones. SKOV3.ip cells were stably transfected with shRNA against Gα12, Gα13, Gαi2 and Gαq. Stable knockdown of these proteins was confirmed by Western blot. (**B**) Effect of silencing different Ga-subunits on LPA-sti\imulated increase in HIF1α levels. The stable cell lines were serum-starved overnight and then stimulated with 10 μM for 4 hours the following day. Immunoblot analysis was carried out with the cell lysates using antibodies to HIF1α followed by stripping and re-probing with antibodies to GAPDH. (**C**) Silencing of Gαi2 abrogates the LPA-stimulated increase in of HIF1α levels. Expression of Gα_i2_ was silenced by siRNA targeting Gαi2 or control siRNA for 48 hrs. Cells were serum starved for 4 hours and then stimulated with 10 μM LPA. After 4 hours, the lysates from the cells were subjected to immunoblot analysis with antibodies to HIF1α. The blot was sequentially stripped and probed with antibodies to Gαi2 and GAPDH equal loading. (**D**) Effect of transient expression of constitutively active mutant of on HIF1α levels. SKOV3.ip (2 × 10^6^) cells were transiently transfected with either pcDNA3 control vector or pcDNA3 vector encoding Gαi2QL, an activated mutant of Gαi2. After 48 hours, the cells were lysed and the lysates were subjected to immunoblot analysis using HIF1α-antibodies. The blot was sequentially stripped and re-probed with antibodies to Gαi2 and GAPDH to monitor Gαi2QL-expression and equal loading respectively (*n* = 3).

Next, we sought to identify the downstream G protein that mediates LPA- signaling in this process. Previous studies from us [5, 7–9, 13, 37] and others [2, 38–41] have shown that LPA-stimulated oncogenic signaling is transduced by the heterotrimeric G protein α-subunits, Gαi2, Gαq, and Gα12/13. Therefore, to identify the G protein involved in LPA signaling to HIF1α, we stably knocked out the expression of individual Gα-subunits, namely Gα12, Gα13, Gαi2, or Gαq in SKOV3.ip cells (Figure 2A) and stimulated these cells with 10 μM LPA for 4 hours and monitored the expression levels of HIF1α. Results from such analysis indicated that the Gαi2-silenced cells showed a marked decrease in HIF1α levels compared to the control cells. In contrast, the silencing of Gα12, Gα13, or Gαq failed to have such an effect (Figure 2B). This was further corroborated using SKOV3.ip cells in which the expression of Gαi2 was transiently silenced using Gαi2-specific siRNAs. As shown in Figure 2B, the ability to induce the expression levels of HIF1α was drastically reduced cells in which the expression of Gαi2 was silenced. To further confirm that Gαi2 is involved in stimulating an increase in HIF1α levels, we transiently transfected SKOV3.ip cells with a constitutively active form of Gαi2 (Gαi2Q205L). The expression of HIF1α was monitored at 48 hrs following the transient expression of the constitutively active Gαi2. As presented in Figure 2C, overexpression of constitutively active Gαi2, without any exogenous LPA, resulted in an increase in HIF1α levels, suggesting that Gαi2-signaling is sufficient and responsible for mediating the effect of LPA in increasing the levels of HIF1α levels in ovarian cancer cells.

### LPA induces EMT in ovarian cancer cells

**Figure 3:**
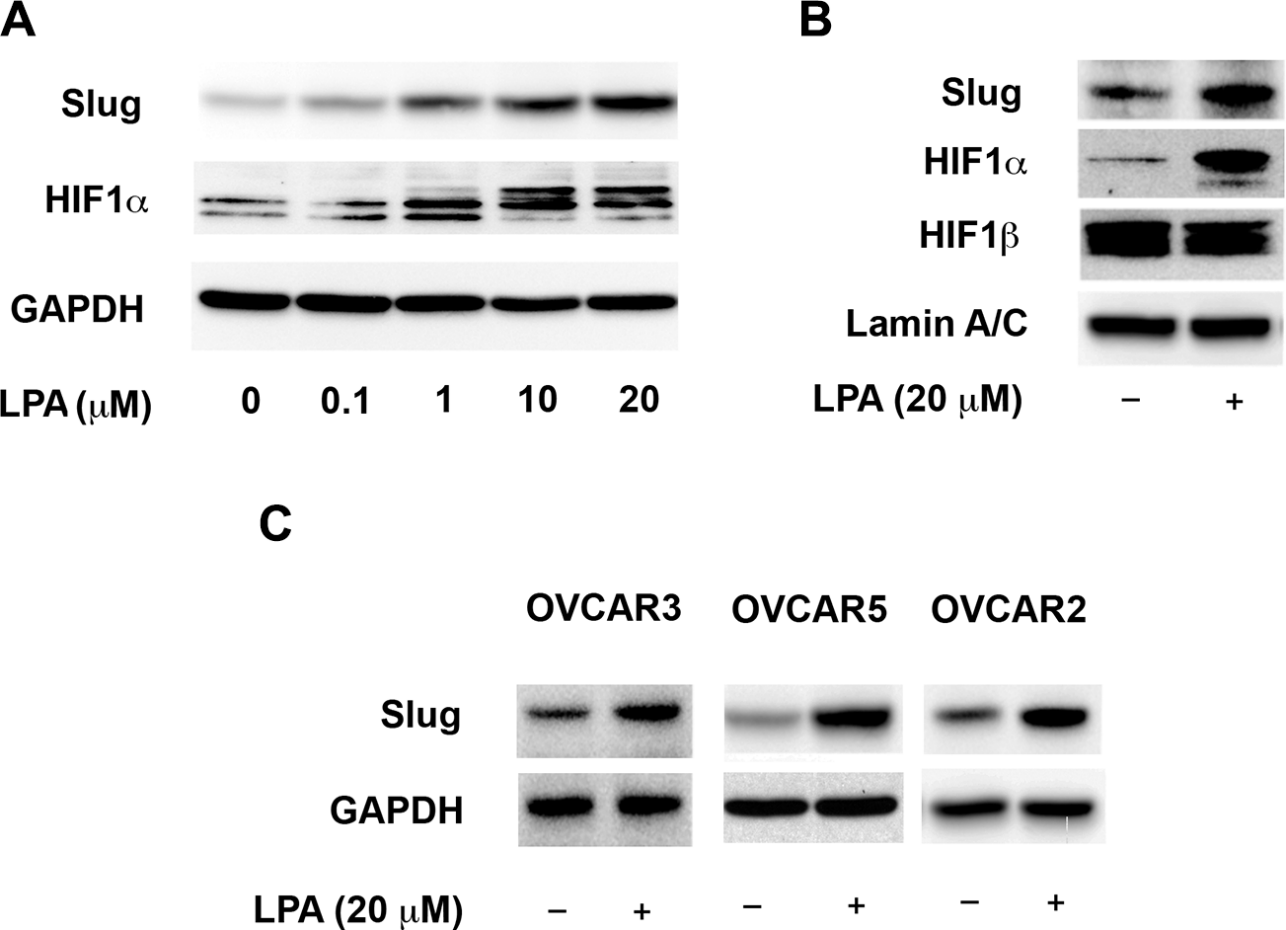
LPA-signaling activates the transcription factors Slug and HIF1α (**A**) HIF1α- and Slug-levels shows a dose-dependent response to LPA stimulation. SKOV3.IP cells were serum starved overnight and then stimulated with the indicated levels of LPA for 4 hours. Cells were lysed and the levels of HIF1α and Slug in the lysates were analyzed by immunoblot analysis using the respective antibodies. The blot was stripped probed with GAPDH-antibodies to monitor equal loading of proteins. (**B**) LPA stimulates the nuclear localization of HIF1α and Slug. SKOV3.IP cells were serum starved overnight and then treated with 20 μM of LPA for 4 hours along with untreated control. Nuclear extracts derived from these cells were subjected to immunoblot analysis using antibodies specific to Slug, HIF1α and HIF1β. Levels of Lamin A/C were used as a marker for nuclear compartment and loading control for equal protein loading in each lane. (**C**) LPA-stimulated increase in the expression of Slug is cell-type independent. OVCAR3, OVCAR5 an OVCAR2 cells were serum-starved overnight and then treated with 20 μM of LPA for 4 hours. Expression levels of Slug Lysates derived from these cells were analyzed for the expression levels of Slug by immunoblot analysis using antibodies for Slug. Levels of GAPDH were analyzed in the stripped blot to ensure equal loading of proteins. (*n* = 3).

It has been well established that the induction of HIF1α expression and its subsequent dimerization with HIF1β to function as a transcription factor in hypoxic conditions is involved in EMT and migration of many different cancer cell types [25, 27, 42]. Taken together with the observation that LPA stimulates HIF1α, it can be surmised that the activation of HIF1α by LPA could promote EMT in ovarian cancer cells. Therefore, we tested whether LPA could stimulate EMT in these cells. Likewise, it has also been well established that Slug, a critical EMT-specific transcription factor, can be used as a marker to monitor EMT [43, 44]. In addition, previous studies have shown that HIF1α can induce the EMT and expression of Slug in many cancer cells [45–47]. Therefore, we monitored the expression of Slug in response to LPA in ovarian cancer cells to test if LPA activated Slug and induced EMT in these cells. SKOV3.ip cells were stimulated with increasing doses of LPA for 4 hours and the expression levels of HIF1α and Slug were monitored by immunoblot analysis from the lysates derived from these cells. As shown in Figure 3A, the expression of HIF1α as well as Slug increased in these cells in a dose-dependent manner. Next, we sought to confirm that the increased expression of HIF1α and Slug by LPA leads to an increased activation of HIF1α and Slug. Since activated HIF1α and Slug translocates to nucleus, the nuclear levels of HIF1α and Slug are often used as indices of their activation status [48, 49]. Accordingly, to assess the activation of HIF1α and Slug by LPA, we determined the nuclear levels of HIF1α and Slug. SKOV3.ip cells were stimulated with 20 μM LPA or vehicle control for 4 hours, following which the nuclear extracts were isolated from these cells. The levels of HIF1α and Slug in these extracts were monitored by immunoblot analysis. Results indicated that the treatment of ovarian cancer cells with 10 μM LPA led to a dramatic increase of HIF1α and Slug in the nucleus of these cells, thus pointing to the strong activation of these transcription factors by LPA (Figure 3B. To establish that the observed effects of LPA is not cell type-dependent, we examined the ability of LPA to induce the expression of Slug in three different ovarian cancer cells lines. As shown in Figure 3C, stimulation with 20 μM of LPA induced Slug expression in all the tested ovarian cancer cell lines: OVCAR3 and OVCAR5 (representing high-grade serous ovarian cancer cell lines [50, 51]) and OVCAR2 cell lines.

### LPA induced EMT in ovarian cancer cells is dependent on Gαi2 and HIF1α

**Figure 4:**
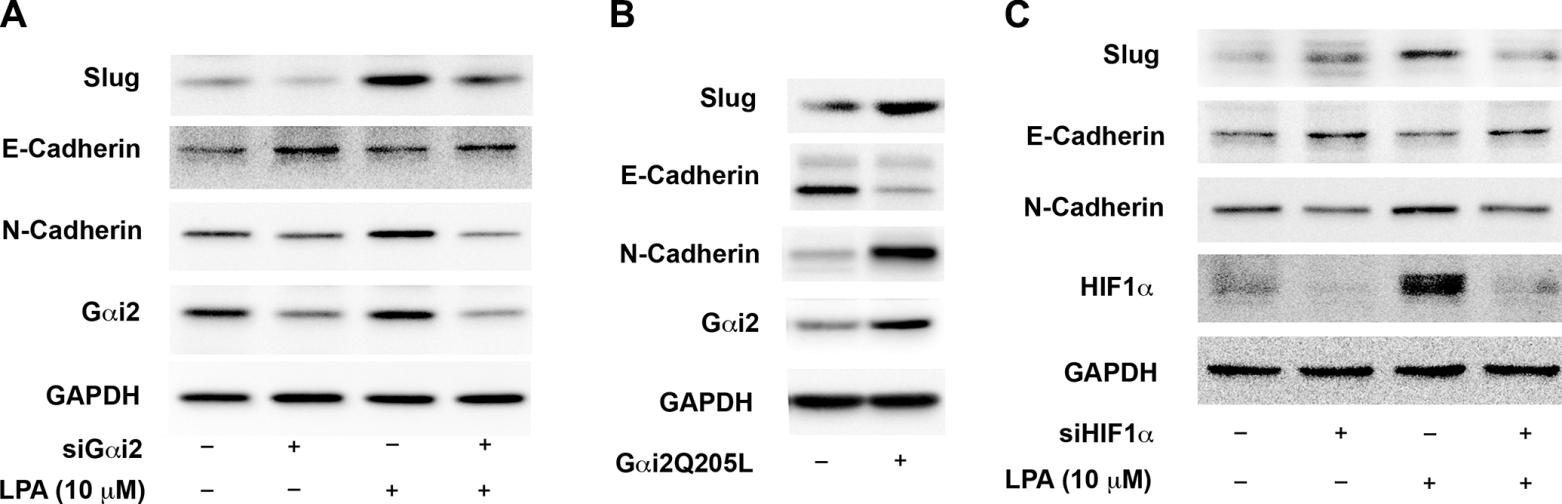
LPA stimulates the expression of EMT markers in ovarian cancer cells via Gαi2 and HIF1α (**A**) Silencing of Gαi2 inhibits LPA-mediated changes in EMT markers. SKOV3.IP cells were transiently transfected with siRNA specific for Gαi2 or with scrambled control siRNA for 48 hours. The cells were serum-starved for 16 hours and then treated with 20 μM of LPA for 4 hours. Lysates from these cells were subjected to immunoblot analysis with antibodies specific to Slug, E-cadherin, and N-cadherin. Silencing of Gαi2 was confirmed by probing the blots with an antibody specific for Gαi2. The blots were stripped and re-probed with antibodies to GAPDH to monitor equal loading of proteins. (**B**) Constitutively active Gαi2 increases the levels of EMT-markers in ovarian cancer cells. SKOV3.IP (2 × 10^6^) cells were transiently transfected with either pcDNA3 vector control or vector encoding the activated mutant of Gαi2, Gαi2QL. After 48 hours, the cells were lysed and the lysates were subjected to immunoblot analysis using antibodies to Slug, E-cadherin, and N-cadherin. The blot was stripped and re-probed with antibodies to Gαi2 and GAPDH to monitor Gαi2QL-expression and equal loading respectively. The experiment was repeated thrice and the results from a typical experiment are presented. (**C**) Silencing of HIF1α inhibits LPA-mediated changes in EMT markers. SKOV3.IP cells were transiently transfected with siRNA directed against HIF1α or with non-targeting siRNA for 48 hours. The cells were serum-starved for 16 hours, following which they were stimulated with 10 μM of LPA for 4 hours. Lysates derived from these cells were subjected to immunoblot analysis using antibodies specific Slug, E-cadherin, and N-cadherin. Silencing of HIF1α was confirmed by using an antibody specific to HIF1α. Levels of GAPDH were assessed to ensure equal loading of each lane. Results are from a typical experiment (*n* = 3).

Our findings presented above (Figures 1–3) indicating the ability of LPA to stimulate the activation of HIF1α via Gαi2 taken together with the established role of HIF1α in the regulation of EMT [27–29] point to a signaling paradigm in which the activation of HIF1α by LPA via Gαi2 is involved in induction of EMT in ovarian cancer cells. To validate such a paradigm, we first analyzed whether the silencing of Gαi2 abrogates LPA-induced expressions of Slug. In addition to Slug, we monitored the anticipated increased expression of N-cadherin and decreased expression of E-cadherin as additional markers for EMT [28, 29]. As shown in Figure 4A, LPA stimulated an increase in the EMT markers N-cadherin and Slug along with a decrease in E-cadherin. The silencing of Gαi2 drastically blunted the ability of LPA to stimulate the increase Slug and N-cadherin as well as its ability to decrease the levels of E-cadherin. The role of Gαi2 in this process was further confirmed using the constitutively activated mutant of Gαi2. SKOV3.ip cells were transiently transfected with a constitutively active Gαi2Q205L and the expression levels of Slug, N-cadherin, and E-cadherin were monitored by immunoblot analysis using the lysates from these transfectants. Consistent with the mediatory role for Gαi2 in this process, the expression of Gαi2QL dramatically increased Slug levels as well as N-cadherin levels with a concomitant decrease in E-cadherin levels (Figure 4B). Since our data demonstrates LPA-Gαi2 signaling axis is involved in the activation of HIF1α (Figure 2), it can be reasoned that the induction of EMT by LPA through Gαi2 involves HIF1α. To establish such a role for HIF1α in LPA-induced EMT in ovarian cancer cells, we tested whether the silencing of HIF1α attenuates the expression of EMT markers in these cells. The expression of HIF1α was silenced in SKOV3.ip cell using HIF1α-specific siRNA. The cells were stimulated with LPA for 4 hours and the expression levels of Slug, N-cadherin, and E-cadherin in the lysates were monitored by immunoblot analysis. As shown in Figure 4C, the silencing of HIF1α in the cells inhibited the increased expression of N-cadherin and Slug along with the decreased expression of E-cadherin, thus validating the conclusion that HIF1α in LPA-induced EMT of ovarian cancer cells is dependent on HIF1α. Together, these results provide strong evidence that the induction of EMT by LPA involves Gαi2-dependent pathway that utilizes downstream transcription factor HIF1α to mediate the induction of EMT.

### LPA enhances hypoxia-induced activation of HIF1α via a Gαi2-dependent pathway

**Figure 5:**
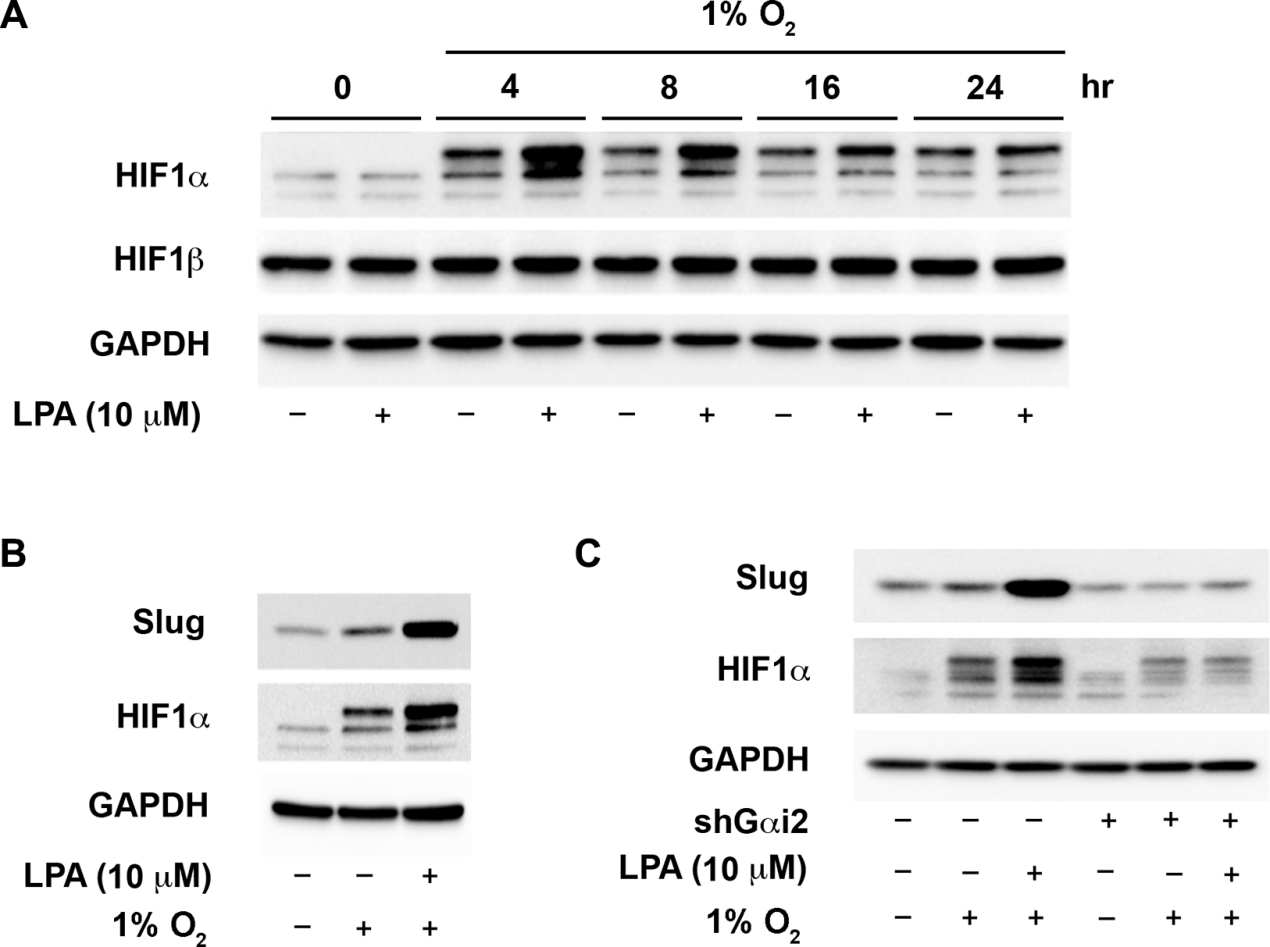
Gαi2 stimulates an increase in the levels of HIF1α in hypoxia (**A**) LPA enhances the up-regulation of HIF1α in hypoxia. SKOV3.ip cells were incubated in a hypoxic chamber containing 1% O_2_ and stimulated with 20 μM of LPA for the indicated lengths of time. Lysates from these cells were analyzed for the levels of HIF1α by immunoblot analysis using the antibodies to HIF1α. The blot was stripped and probed with antibodies to HIF1β and GAPDH to monitor their respective levels. The levels of GAPDH were used to monitor equal loading of proteins. (**B**) LPA enhances hypoxia-mediated increase in Slug levels. SKOV3.ip cells were serum-starved overnight. The control group was left in normoxic conditions while the hypoxia and the hypoxia plus LPA-stimulated cells (20 μM of LPA) were put into the hypoxic chamber (1% O_2_) for 4 hours in serum-free medium. Lysates from these cells were subjected to immunoblot analysis with the antibodies to Slug. GAPDH was probed in the stripped blot to ensure equal loading of proteins in each lane (**C**) Gαi2 is required for the increased expression of HIF1α and Slug in hypoxia. SKOV3.ip cells with either non-sense shRNA or with shRNA that targeted Gαi2 were placed in hypoxic chamber along with normoxic control group. Cells under hypoxia were stimulated with 10 μM of LPA for 4 hours. Lysates derived from these cells were subjected to immunoblot analysis using antibodies to HIF1α and Slug. Levels of GAPDH in the stripped blot were monitored to ensure equal loading of the proteins in each lane. Profile from a representative experiment is presented in each panel (*n* = 3).

Ovarian cancer cells are often found in the hypoxic environment of the peritoneal cavity and the core of the primary tumor [52]. A previous report has demonstrated that exogenous LPA stimulation synergistically enhanced hypoxia-induced stabilization of HIF1α and hypoxia, which in turn, could enhance the oncogenic responsiveness of ovarian cancer cells to LPA [52]. However, the signaling mechanism and the role of G protein(s) in enhancing HIF1α activation remain to be elucidated. Therefore, we set out to determine if LPA signaling could enhance the levels of HIF1α and subsequent EMT of ovarian cancer cells in a hypoxic environment. To test, ovarian cancer cells incubated in 1% oxygen environment were stimulated with 10 μM of LPA for different lengths of time along with untreated controls. As anticipated, hypoxia alone increased the stabilization of HIF1α (Figure 5A). However, HIF1α levels were markedly increased when these cells were stimulated with LPA (Figure 5A). Similar to the results found in normoxic conditions, HIF1α was maximally stabilized at 4 hours in hypoxic conditions. Next, we examined the effect of hypoxia alone or LPA plus hypoxia on the levels Slug, N-cadherin and E-cadherin. While hypoxic conditions alone induced HIF1α stabilization and up-regulation of Slug, stimulation of these cells with exogenous LPA dramatically enhanced the up-regulation of Slug and HIF1α compared to hypoxic condition alone (Figure 5B). Furthermore, exogenous LPA drastically down-regulated the expression of E-cadherin. Overall, these data points to the synergistic role of LPA in enhancing the responsiveness of ovarian cancer cells to hypoxia and inducing EMT. Hypothesizing that the synergistic effect elicited by LPA could involve the Gαi2-dependent mechanism, identified in normoxic conditions, we investigated whether the silencing of Gαi2 abrogates such LPA-stimulated synergistic effect on the hypoxic response involving HIF1α and Slug. SKOV3.ip cells in which the expression of Gαi2 was silenced were incubated in hypoxic condition and stimulated with 10 μM LPA along with untreated controls. Our results indicated that the silencing of Gαi2 blunted the ability of LPA to enhance the expression of both Slug and HIF1α in hypoxic conditions (Figure 5C). Remarkably, silencing Gαi2 alone, with no exogenous LPA present, led to decreased levels of HIF1α and Slug compared to controls in hypoxic conditions with no exogenous LPA. This suggests that Gαi2 is necessary for the induction of HIF1α in hypoxic conditions (Figure 5C). Finally, to confirm that Gαi2 is needed for hypoxia-induced EMT, both in the presence and absence of exogenous LPA, we analyzed the levels of E-cadherin and N-cadherin in response to LPA in ovarian cancer cells incubated in hypoxia or normoxia. As shown in Figure 5D, silencing of Gαi2 inhibited down-regulation of E-cadherin and up-regulation of N-cadherin indicating that Gαi2 is necessary for inducing EMT in hypoxic conditions both with and without exogenous LPA.

### LPA stimulates the activation of Src via Gαi2

**Figure 6:**
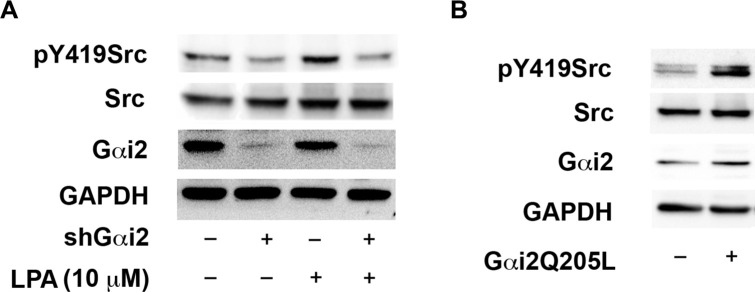
LPA stimulates the activation of Src via Gαi2 (**A**) LPA-mediated activation of Src involves Gαi2. SKOV3.ip cells were transiently transfected with either nonsense shRNA or shRNA that targeted Gαi2. After 24 hours, the transfected cells were serum starved for 16 hours and treated with 10 μM of LPA for 20 minutes along with untreated control groups. Lysates were subjected to immunoblot analysis using antibodies specific to Src phosphorylated on Tyr-419. The blot was stripped and probed for total Src to monitor expression levels and equal loading of proteins. A parallel blot was used to monitor for the silencing of Gαi2 using antibodies specific to Gαi2. The blot was stripped and re-probed with antibodies specific to GAPDH to monitor equal loading of proteins. (**B**) Constitutively activated Gαi2 stimulates Src. SKOV3.ip (2 × 10^6^) cells were transiently transfected with either vector control or with constitutively active Gαi2QL. After 48 hours, the transfectants were lysed and subjected to immunoblot analysis with antibodies specific to tyrosine-419 phosphorylated Src. The blot was stripped and re-probed for total Src. Expression of constitutively active Gαi2 was confirmed with antibodies specific to Gαi2. The blots were stripped and re-probed with antibodies to GAPDH to monitor equal loading of proteins. Presented results are from a typical experiment (*n* = 3).

Recent reports from our lab [8, 15] have indicated that Src, via Gαi2, is involved in initiating invasive migration of ovarian cancer cells. Additionally, Src has been shown to activate HIF1α by diverse pathways involving both direct as well as indirect mechanisms [53–55]. Therefore, we investigated if Src is involved in activating HIF1α and Slug. First, to confirm that Gαi2 is needed for the activation of Src by LPA, we transiently silenced Gαi2 in these cells and stimulated with 10 μM LPA. Activation of Src was monitored by the phosphorylation status of Src on tyrosine residue 419, which has been established as a good indicator of Src activation [8]. As shown in Figure 6A, silencing Gαi2 inhibited the activation of Src, indicating that Gαi2 is necessary for the activation of Src by LPA. To confirm that Gαi2 is sufficient to activate Src, we transiently transfected ovarian cancer cells with constitutively active Gαi2, without any exogenous LPA stimulation, and checked the phosphorylation of Tyr-419 of Src. As shown in Figure 6B, constitutively active Gαi2 led to the activation of Src indicating the ability of Gαi2 to activate Src.

### LPA induces EMT of ovarian cancer cells via Gαi2 and Src

**Figure 7:**
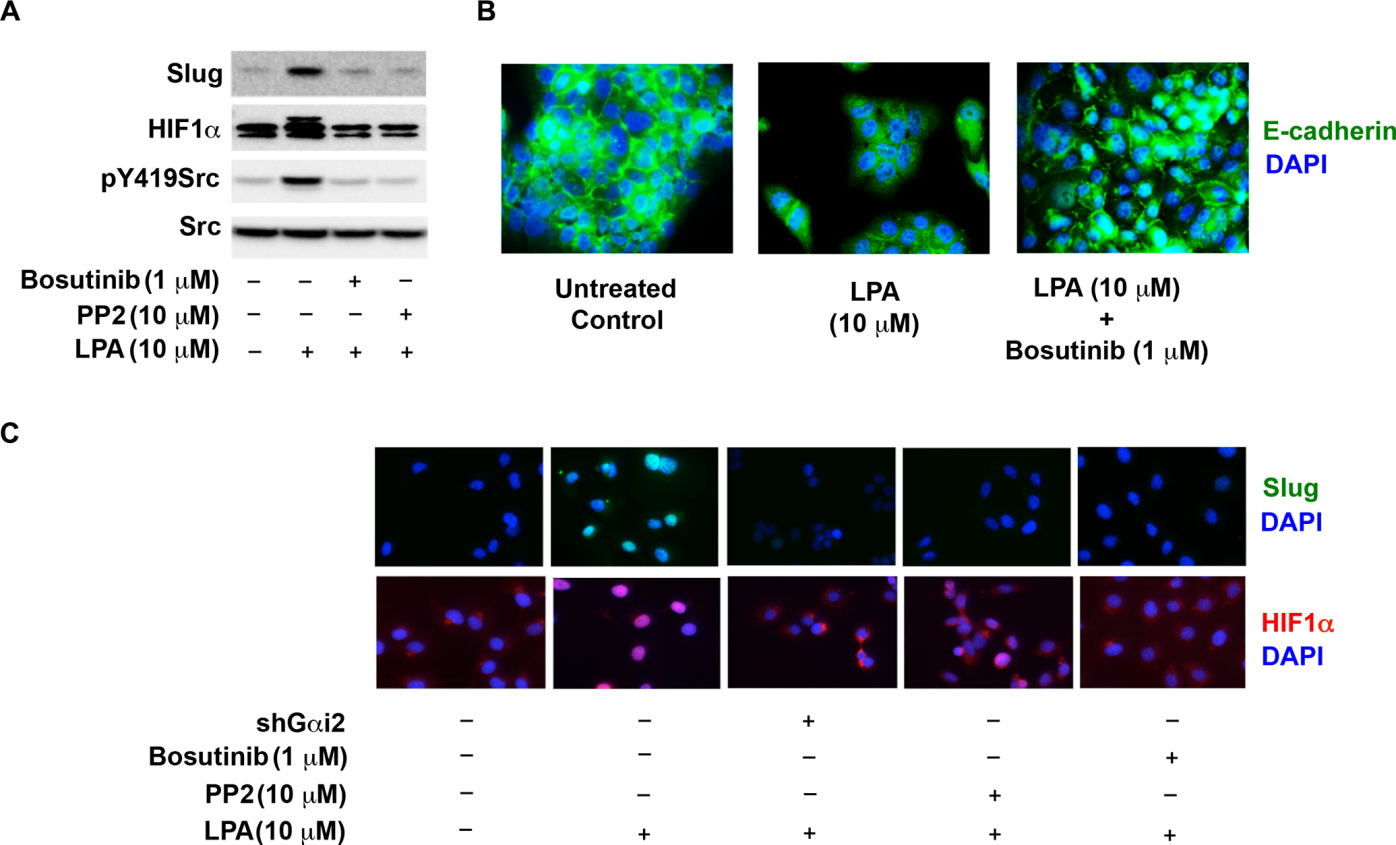
Src is required for the expression and activation of HiF1α and Slug (**A**) Src is required for LPA-mediated increase in the levels of HIF1α and Slug. SKOV3.ip cells were stimulated with 10 μM LPA with or without the incubation with 10 μM PP2 or 1 μM Bosutinib for 4 hours. Lysates from these cells were subjected to immunoblot analysis using antibodies to HIF1α, Slug, pY419 Src and total-Src. (**B**) Knockdown of Gαi2 or inhibition of Src ablates LPA-stimulated activation of HIF1α and Slug. Parental SKOV3.ip cells, SKOV3.ip cells in which Gαi2 using specific shRNA, SKOV3.ip cells treated with 10 μM PP2, or SKOV3.ip cells treated with 10 μM Bosutinib were stimulated with 10 μM LPA for 4 hours along with the unstimulated control. Cells were stained with an antibody against Slug or HIF1α and counterstained with DAPI. (**C**) Inhibition of Src attenuates LPA-induced EMT. OVCA432 cells were stimulated with 10 μM LPA for 4 hours or pre-treated 1 μM Bosutinib prior to stimulation with 10 μΜ LPA for 4 hours along with untreated control group. At 4 hours cells were stained with an antibody against E-cadherin and counterstained with DAPI (*n* = 3).

Next, we investigated whether Src is involved in activating HIF1α and Slug. To test SKOV3.ip cells were treated with PP2, an inhibitor of the Src family of kinases, [53, 56], or Bosutinib, a clinically used Src inhibitor [57]. These cells were stimulated with LPA and the expression levels of HIF1α and Slug were monitored. The efficiencies of the inhibitors were monitored by he phosphorylation status of Tyr-419 of Src by immunoblot analysis. As shown in Figure 7A, treatment with PP2 and Bosutinib significantly inhibited the increase in the levels of Slug and HIF1α, suggesting that Src is required for the effect of LPA on HIF1α- and Slug-levels. Next, we investigated whether Gαi2 and Src were involved in the activation of Slug and HIF1α. The translocation of Slug and HIF1α to the nucleus has been used as an indicator of their respective activation. Therefore, we carried out immunofluorescence microscopic analysis to demonstrate the translocation of Slug (Figure 7B) and HIF1α (Figure 6E) to the nucleus. Treatment of SKOV3.ip cells with 10 μM of LPA led to translocation of Slug and HIF1α to the nucleus, indicating the activation of these transcription factors. More importantly, our results demonstrated that the stable knockdown of Gαi2 or treatment of cells with either of the Src-inhibitors, PP2 or Bosutinib, significantly reduced the translocation of Slug (Figure 6D) and HIF1α (Figure 7B) to the nucleus in reponse to LPA. Together, these results indicate that LPA stimulates Gαi2, which then utilizes Src to activate both Slug and HIF1α, the mediators of EMT. To demonstrate the role of Src in LPA-induced EMT, we utilized immunofluorescence analysis of OVCA432 in response to exogenous LPA. OVCA432 cells have previously been shown to be a good model system for studying EMT in ovarian cancer cells due to fact that these cells express high levels of E-cadherin and have more of an epithelial phenotype compared to other ovarian cancer cell lines [58]. As shown in Figure 7A, OVCA432 cells in serum-free media demonstrated a distinct epithelial phenotype with strong E-cadherin staining between the cells. Conversely, treatment of OVCA432 cells with LPA overnight significantly decreased the overall expression levels of E-cadherin (Figure 7A, middle panel). In addition a drastic reduction in the presence of E-cadherin levels in cellular junctions could be observed in these cells. Interestingly, treatment of OVCA432 cells with Bosutinib drastically reduced the ability of LPA to down-regulate E-cadherin localization in inter-cellular junctions as well as its overall expression, indicating that the blockade of Src activation prevented LPA-induced EMT and that Src is needed for induction of EMT of ovarian cancer cells.

### Inhibition of HIF1α attenuates LPA-induced EMT and cell migration

**Figure 8:**
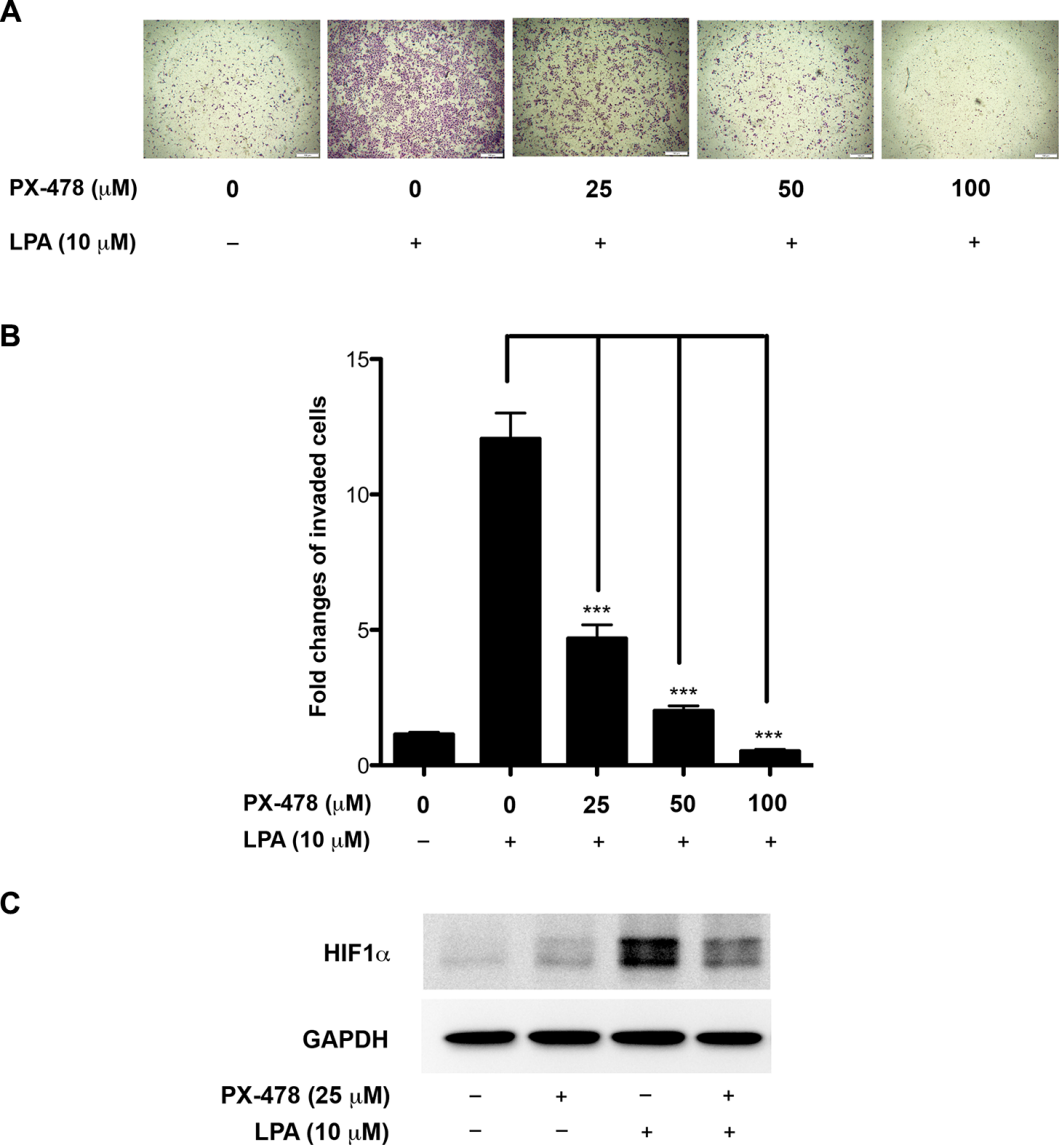
Inhibition of HIF1α attenuates LPA-induced invasive-migration of ovarian cancer cells SKOV-ip cells were stimulated with LPA or LPA plus varying concentration of HIF1α inhibitor PX-478 for 16 hours. A transwell invasive migration was carried out as detailed under Materials and Methods following our previously published procedure. Representative micrograph images of Hemacolor stained invaded cells were obtained at 100× for each of the experimental groups are presented (**A**). Migrated cells were quantified and presented as fold change over untreated control values (**B**). Immunoblot analysis with antibodies to HIF1α was carried out to verify the inhibitory effect of PX-478 on HIF1α expression levels (**C**). Representative data from a typical experiment is presented (*n* = 3; ***p < 0.0001).

Since EMT has been shown to promote invasive migration of cancer cells [28, 59], our results would suggest that the stimulation of HIF1α by LPA is required for such invasive migration of ovarian cancer cells. It has been shown that PX-478, a clinically used inhibitor of HIF1α, attenuates the activity of HIF1α by lowering its expression levels [60, 61]. Therefore, we tested whether LPA-stimulated invasive migration of ovarian cancer cells could be attenuated by the inhibition of HIF1α by PX-478. As shown in Figure 7, LPA potently stimulated the invasive migration of SKOV3.ip cells. However, treating these cells with the escalating doses of PX-478 led to a concentration-dependent inhibition of invasive migration (Figure 8A and 8B). Even at the lowest tested dose of 25 μM concentration - at which the PX-478 markedly reduced the cellular levels of HIF1α (Figure 8C) -, PX-478 attenuated the invasive migration of SKOV3.ip cells (Figure 8A and 8B). Together, our findings establish the functional role for LPA-Gαi2-Src stimulated HIF1α in promoting EMT phenotype in ovarian cancer cells involving the overexpression of Slug and increased invasive migration.

## DISCUSSION

Previously, we have shown the critical role of LPA in promoting cell proliferation and migration in ovarian cancer cells [7–9, 15] in addition to establishing the role of downstream Gαi2, Gα12, and Gα13 in ovarian cancer xenograft growth *in vivo* [14]. In the present study, we demonstrate the role of LPA in the potent activation of HIF1α in ovarian cancer cells. Using a transcription factor reporter array, we show that LPA stimulates the activation of HIF1α by 150-fold within 20 minutes (Figure 1). Further analysis of the underlying mechanism indicates that the activation of HIF1α by LPA involves Gαi2-dependent signaling mechanism (Figure 2) that can be correlated with the increased expression of Snail, which is involved in promoting the transcriptional activation of EMT-specific genes (Figures 3 and 4). Our results further demonstrate that the presence of LPA synergistically increases the expression levels of HIF1α through a Gαi2-dependent signaling pathway in hypoxic conditions, such as those found in the ascites fluid of ovarian cancer patients. In this context, we demonstrate here that silencing Gαi2 alone, completely of abrogates hypoxia-induced expression of HIF1α even in the absence of exogenous LPA. These results indicate that Gαi2 is required for the hypoxia-induced expression of HIF1α in ovarian cancer cells (Figure 5). Interrogating further, we establish that Gαi2-dependent signaling involves Src to activate HIF1α (Figure 6). LPA-stimulated signaling nexus involving Gαi2 and Src, thus formed, induces EMT in ovarian cancer cells as indicated by the nuclear translocation of Slug and up-regulation of N-cadherin expression levels and loss of E-cadherin between cells (Figure 7). Finally, we demonstrate that the inhibition of this pathway using PX-478, a HIF1α inhibitor, drastically decreases the migration of ovarian cancer cells (Figure 8). Thus, Our results presented here demonstrate for the first time that LPA signaling in normoxic conditions activates a Gαi2-Src-dependent pathway to up-regulate the transcription factors HIF1α and Slug and the demonstrated Gαi2-Src pathway is critical for induction of EMT by LPA. Importantly, we show that Gαi2 is necessary for hypoxia-induced activation of HIF1α and that LPA, via a Gαi2-Src-dependent signaling, synergistically enhances hypoxia-induced activation of HIF1α and Slug.

**Figure 9:**
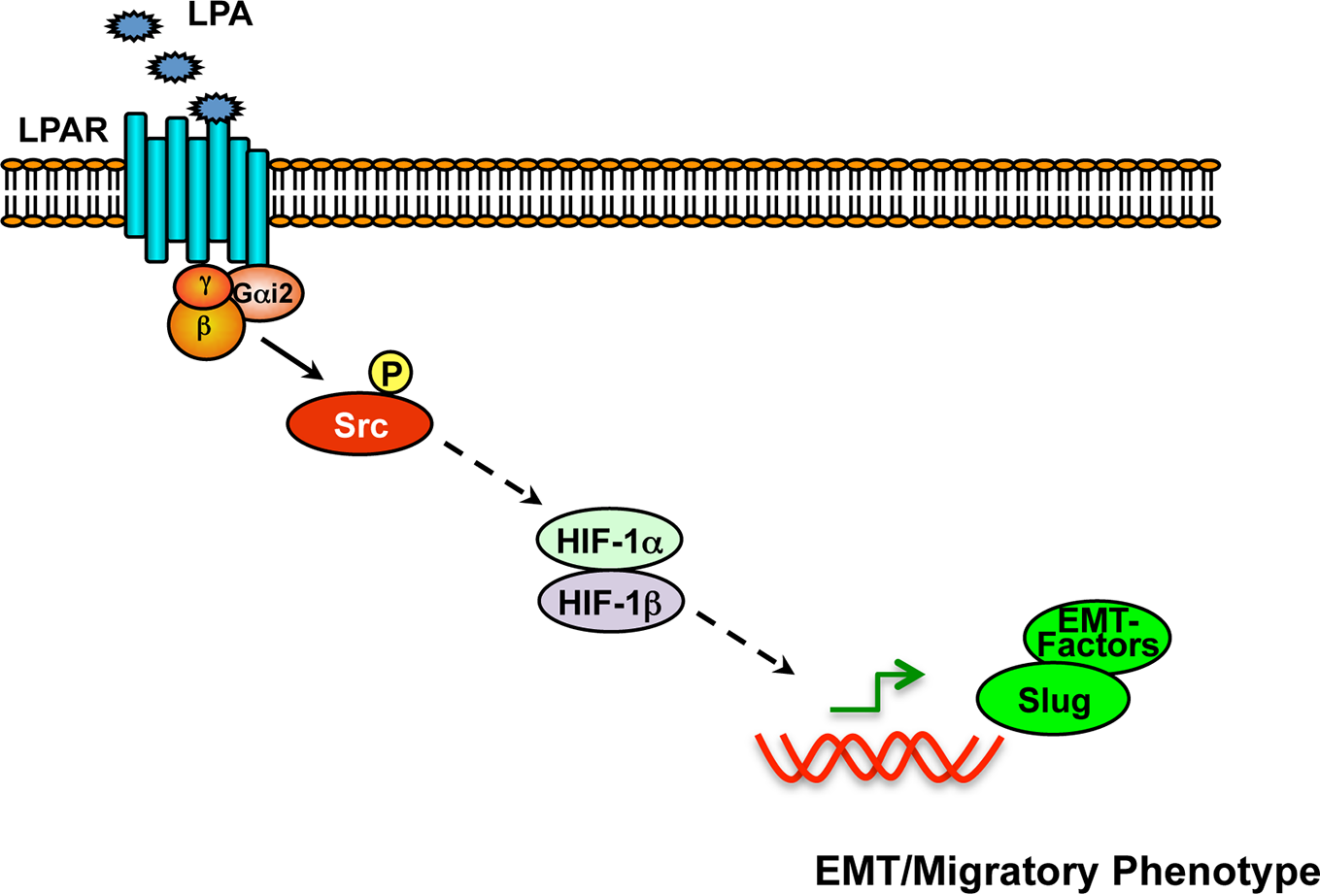
Schematic representation of Gαi2–Src-HIF1α nexus in the regulation of EMT in ovarian cancer cells Stimulation of LPA receptors leads to the activation of Gαi2 and the subsequent activation of Src, as we have shown previously [8, 15]. Src-dependent signaling, in turn, stimulates the upregulation and activation of HIF1α. HIF1α, once activated, stimulates the expression and resultant activation of Slug and other EMT-specific factors including N-cadherin, thereby promoting EMT and associated invasive migration of cancer cells.

Previous studies have shown that the levels of HIF1α is regulated at multiple levels such as the inhibition of degradation of HIF1α, increased translation of HIF1α mRNA, and enhanced transcription of HIF1α gene [62]. Although these mechanism are not mutually exclusive, the observation that the effect of LPA on the activation of HIF1α can be seen by as early as 20 minute, points to the role of LPA in the stabilization of HIF1α through Gαi2. A novel and yet another critical observation reported here is the finding that the stabilization of HIF1α in hypoxic condition - independent of exogenous LPA treatment - is also dependent on Gαi2. Previous findings from our laboratory have indicated that LPA-Gαi2 signaling could rapidly stimulate Rac via p130Cas/Src dependent pathway [15]. It has also been shown that Src can stimulate an increase in the levels and subsequent activation of HIF1α involving Rac-stimulated ROS generation via NADPH oxidase [54]. Connecting these two independent observations, our data presented here points to a signaling paradigm in which the signaling by LPA propagates through Gαi2 and Src to HIF1α (Figure 9).

HIF1α signaling has been linked to EMT and cancer progression. There is voluminous reports that HIF1α and hypoxic conditions are linked to EMT [27]. Indeed, HIF1α has been linked to directly up-regulating the expression of Twist [63, 64] and Snail [65]. Similarly, several very recent reports have shown that HIF1α can induce expression of Slug [45–47]. A recent has shown that knockdown of HIF1α resulted in decreased mRNA levels of Slug, indicating that HIF1α is directly or indirectly involved in Slug expression [45]. It has also been shown that Slug is involved in the transcriptional repression of E-cadherin [66, 67]. In this context, our current study defines the upstream signaling mechanism involving a specific G protein in the activation of HIF1α and subsequently Slug. Future studies should define the mechanism by which HIF1α increases the transcription of Slug. Nevertheless, it is clear that HIF1α and Slug are two transcription factors whose levels are directly increased by LPA via the Gαi2-Src signaling node. These findings provide evidence that this signaling node can be targeted directly to inhibit expression of Slug and stabilization of HIF1α. Since these two transcription factors have been shown to be important in EMT and drug resistance in a multitude of cancers, our findings underscore the possibility that the pathway we have identified here is directly contributing to ovarian cancer progression and potentially drug-resistance. Recent finding that the expression of Gαi2 increases in advanced stage ovarian cancers [68], further points to critical role of Gαi2 and the identified pathway as a potential therapeutic signaling node in advanced ovarian cancer. Moreover, besides contributing to cell migration and EMT, it is highly likely that HIF1α activation via the Gαi2-Src pathway is also involved in other effects such as resistance to apoptosis, enhanced glucose uptake, and angiogenesis, all of which have been shown to be critically involved in tumor growth and progression.

Of critical importance, we show here that Gαi2 is also necessary for HIF1α activation independent of exogenous LPA signaling. Thus, there is a distinct possibility that LPA and/or other ligands that utilize Gαi2, such as CXCL12, could be responsible for activation HIF1α in hypoxic conditions via autocrine/paracrine signaling. However, this needs to be investigated further. Nonetheless, our report demonstrates that Gαi2 and Src are needed for HIF1α activation in hypoxic conditions, indicating that inhibition of this pathway can suppress hypoxia-induced resistance in ovarian cancer patients. This is also the first report to our knowledge that has shown the importance of LPA-signaling via Gαi2 in inducing EMT. Although a recent study reported the ability of LPA to induce EMT via Wnt/β-catenin signaling pathways [69], the underlying mechanism was not fully clarified. In this regard, our study presented here firmly establishes the role of Gαi2-Src-HIF1α signaling nexus in promoting LPA-stimulated induction of Slug which is involved in EMT. Finally, this report adds to our previous findings [8, 15] that demonstrated the role of Gαi2-Src-p130Cas-dependent mechanism in LPA-induced invasive-migration of ovarian cancer cells. It should be noted here that PX-478 has been shown to enhance the anti-tumor effects of both radio- as well as chemotherapeutic modalities [61, 70–72]. Based on the potent inhibitory effect of PX-478 on HIF1α levels, one can speculate that the treatment with PX-478 will downregulate the multitudes of HIF1α-regulated genes, including those involved in EMT phenotype such as Slug and resultant ddecrease in the expression of E-cadherins. In this context, our present observation that the clinically relevant dose of PX-478 (25 μM) potently inhibits the invasive migration of ovarian cancer cells (Figure 8) further establishes the therapeutic potential of the LPA-Gai-HIF1α signaling node (Figure 9), especially in HIF1α overexpressing ovarian cancers.

## MATERIALS AND METHODS

### Cells and reagents

The ovarian cancer cell lines OVCAR2, OVCAR3, OVCAR5 and OVCA432 were kindly provided by Susan K. Murphy (Duke University, Durham, NC). SKOV3.ip1 cells (SKOV3.ip), an *in vivo* passaged variant of SKOV3 cells established by Yu et al., [73] were kindly provided by Dr. Robert C. Bast (MD Anderson Cancer Center, Houston, TX). OVCAR2, OVCAR3, OVCAR5, OVCA429, OVCA432 and SKOV3.ip cells were maintained in Roswell Park Memorial Institute (RPMI) 1640 media (Mediatech, Manassas, VA) containing 10% FBS (Gemini Bio-Products, West Sacramento, CA), 50 μ/mL penicillin, 50 mg/mL streptomycin (Mediatech, Manassas, VA) at 37°C in a 5% CO_2_incubator. For serum-starvation, the media used was RPMI 1640 with 0.1% BSA (Roche, Indianapolis, IN), 50 U/mL penicillin and 50 mg/mL streptomycin (Mediatech). Lysophosphatidic acid (1-oleoyl-2-hydroxy-sn-glycero-3-phosphate) was obtained from Avanti Polar Lipids (Alabaster, AL) and dissolved into 10 mM stock solutions in PBS with 0.1% BSA and stored at −80°C until use. Non-target control shRNA pLKO.1 vector construct was purchased from Sigma-Aldrich, St. Louis, MO (SHC002) whereas pLKO.1 vector constructs targeting Gαi2 (RHS3979-9596925) was purchased from Open Biosystems (Lafayette, CO). The siGENOME Non-targeting siRNA (D-001206-13-05), siGENOME SMARTpool Gαi2 (M-003897-00-0005) and HIF1α (M-004018-05-0005) were purchased from Dharmacon (Lafayette, CO). Peroxidase-conjugated anti-rabbit IgG was purchased from Promega (Madison, WI), and peroxidase-conjugated anti-mouse was purchased from GE Healthcare (Little Chalfont, UK). E-cadherin antibody was purchased from Santa Cruz Biotechnology (Santa Cruz, CA). HIF1α antibody was purchased from BD Biosciences (San Jose, CA). Alexa 568 anti-mouse and Alexa 488 anti-rabbit antibodies were purchased from Invitrogen (Eugene, OR). DAPI was purchased from Life Technologies and used at a working concentration of 0.25 μg/mL.

### Cell transfection

All cells were transfected with a Nucleofector II system from Lonza (Allendale, NJ) using the provided transfection protocol for SKOV3 cells as published previously [8, 15]. SKOV3.ip cells were trypsinized and counted using a Countess automated cell counter (Life Technologies). 2 × 10^6^ cells per transfection cuvette were transfected with either non-targeting siRNA (100 nM), siRNA targeting Gαi2 (100 nM), Gαi2QL (2 μg) or pcDNA3 vector (2 μg) as indicated. After transfection, the cells were plated on 60 mm plates and allowed to adhere overnight. The following day the media was changed and the cells were allowed to grow until the end of the day. The cells were then were re-plated at a density of 5 × 10^5^ cells per 100 mm plate and allowed to adhere overnight. For stable transfection, SKOV3.ip cells transfected as previously described with shGαi2 or control, nonsense shRNA and selected for the expression of shGαi2 or the nonsense vector with puromycin [9].

### Hypoxia treatment

Hypoxia treatments (1% O_2_) were performed in INVIVO_2_ 400 hypoxia workstation (Baker, Sanford, ME). Cells were incubated with 5% CO_2_ and 1% O_2_ at 37°C for the indicated lengths of time. After incubation, cells were collected and western blot analysis was carried out.

### Transcription factor reporter assay

Cignal^™^ 45-Pathway Reporter Arrays (Qiagen, CA) was used to screen for different transcription factors upon LPA stimulation of SKOV3.ip ovarian cancer cells. Cells were seeded into wells (50,000 cells/well) of the Cignal^™^ Finder 96-well plates (Qiagen, CA) to transfect the reporters into cells via reverse transfection according to manufacturer's protocol. Briefly, reporter DNA constructs resident in each well of the plate were resuspended with 125 μl Opti-MEM and complexed with 25 μl of Lipofectamine 2000 (ThermoFisher, CA) transfection reagent. Each well is added with 5 × 10^4^ cells suspended 25 μl of Opti-MEM media. Transfection was allowed to happen by incubating the plate for 24 h at 5% CO_2_ and 37°C. Following transfection, the cells were serum deprived for 16 h and treated with either vehicle (0.1% BSA in PBS) or LPA (20 μM) for 20 min. Differential activation of the transcription factors were determined by lysing the cells and measuring the luminescence intensity following the manufacturer's protocol.

### Fluorescence imaging

OVCA432 and SKOV3.ip cells were plated at density of 1 × 10^5^ in 6-well plates with glass coverslips at the bottom. The cells were allowed to adhere overnight in a 37°C incubator with 5% CO_2_. The cells were washed 3× with sterile PBS and then serum-starved for 4 hours. After serum-starvation, the cells were treated with 10 μM of LPA for 4 hours. After LPA treatment, the cells were washed with ice-cold PBS one time and then treated with 4% paraformaldehyde for 15 minutes while rocking. The cells were then washed with PBS 1× and then stored at 4°C until they were stained. All treatment groups were lysed with 0.25% Triton X-100 for 10 minutes and then washed with PBS 3×. After washing, the coverslips were blocked with 1% BSA in PBS for 30 minutes at room temperature while rocking. After blocking, the coverslips were washed with PBS 1×. After washing, the primary antibody was applied in 1%-BSA in PBS and rocked for 10 minutes at room temperature. The coverslips were then transferred to 4°C and incubated overnight while rocking. The following day the primary antibody was removed and the coverslips were washed 3× for 5 minutes each. After washing, the coverslips were incubated with fluorescently tagged secondary antibody for 45 minutes at room temperature while rocking and covered with aluminum foil. After incubation with the secondary antibody, the coverslips were washed 1× with PBS and then stained with DAPI for 5 minutes. The coverslips were then washed 3× with PBS for 5 minutes each wash and then allowed to dry. Once dry, the coverslips were mounted with ProLong Gold antifade from Life Technologies (Grand Island, NY) on glass slides. The coverslips were allowed to dry overnight at room temperature in the dark and then imaged the following day with a Nikon Eclipse Ni-U (Melville, NY) at 600×.

### Collagen-1 transwell migration assay

The Collagen-1 migratory invasion assay was performed as previously published [8]. Collagen type 1 was coated overnight onto 8-mm pore transwells at 4°C. The following day, the collagen-coated cell culture inserts containing 5 × 10^4^ SKOV3.ip cells were suspended in 200 μL serum-free media were placed in the well of a 24-well companion plate. Each well contained 500 mL media containing serum-free media control or serum-free media containing 10 μM of LPA. The cells were incubated for 20 hours. Non-migrating cells on the proximal side of the inserts were removed with a cotton swab, and the migrated cells on the distal side of the inserts were fixed and stained with Hemacolor (EMD Chemicals). Images were obtained at 100× in 5 random fields for each group. The experiments were repeated 3 times. SKOV3.ip cells were transfected with the indicated plasmid (shRNA) and plated into 6-well plates for a total of 48 hours. Twenty-four hours after transfection, the cells were serum starved for an additional 24 hours, trypsinized, counted, and placed into the transwell.

### Western blotting

Immunoblot analysis with the indicated antibodies were carried out following previously published procedures [8, 74] and developed with a Kodak Image Station 4000 MM.

### Statistical analysis of data

An unpaired two-tail *t*-test with Welch's correction was performed to determine statistical significance.

## Abbreviations

LPA: lysophosphatidic acid
HIF1: hypoxia-induced factor-1
EMT: epithelial-to-mesenchymal transition
